# Synergistic Association of PTGS2 and CYP2E1 Genetic Polymorphisms with Lung Cancer Risk in Northeastern Chinese

**DOI:** 10.1371/journal.pone.0039814

**Published:** 2012-06-25

**Authors:** Shujie Guo, Xiaobo Li, Min Gao, Hong Kong, Yuqiong Li, Mingliang Gu, Xiaoqun Dong, Wenquan Niu

**Affiliations:** 1 State Key Laboratory of Medical Genomics, Ruijin Hospital, Shanghai Jiao Tong University School of Medicine, Shanghai, China; 2 Department of Respiratory Medicine, The First Affiliated Hospital of Harbin Medical University, Harbin, Heilongjiang, China; 3 Beijing Institute of Genomics, Chinese Academy of Sciences and Key Laboratory of Genome Science and Information, Chinese Academy of Sciences, Beijing, China; 4 Department of Biomedical and Pharmaceutical Sciences, College of Pharmacy, The University of Rhode Island, Kingston, Rhode Island, United States of America; Roswell Park Cancer Institute, United States of America

## Abstract

**Background:**

Lung cancer is the most common cause of cancer-related deaths worldwide. The aim of this study was to investigate the association of five extensively-studied polymorphisms in *PTGS2* (rs689466, rs5275, rs20417) and *CYP2E1* (rs2031920, rs6413432) genes with lung cancer risk in a large northeastern Chinese population.

**Methodology/Principal Findings:**

This is a hospital-based case-control study involving 684 patients with lung cancer and 604 cancer-free controls. Genotyping was performed using the PCR-LDR method. Data were analyzed using Haplo.stats and MDR programs. There were significant differences between patients and controls in allele/genotype distributions of rs5275 (*P* = 0.002/0.003) and rs6413432 (*P* = 0.037/0.044), as well as in genotype distributions of rs689466 (*P* = 0.02). The risk for lung cancer associated with the rs5275-C mutant allele was decreased by 60% (95% CI [confidence interval]: 0.21–0.74; *P* = 0.004) under the recessive model. Carriers of rs689466-G mutant allele had a 28% (95% CI: 0.57–0.92; *P* = 0.008) reduced risk of developing lung cancer relative to the AA genotype carriers. In haplotype analysis, haplotype G-C-C-T (in order of rs689466, rs5275, rs2031920 and rs6413432) decreased the odds of lung cancer by 28% (95% CI: 0.51–0.93; *P* = 0.019) after adjusting for confounding factors, whereas haplotype A-T-T-T had 1.49-fold (95% CI: 1.21–1.79; *P* = 0.012) increased risk for lung cancer. Using MDR method, the overall best model including rs5275, rs689466 and rs6413432 polymorphisms was identified with a maximal testing accuracy of 66.1% and a maximal cross-validation consistency of 10 out of 10 (*P* = 0.003).

**Conclusions/Significance:**

Our findings demonstrated a potentially synergistic association of *PTGS2* and *CYP2E1* polymorphisms with the underlying cause of lung cancer in northeastern Chinese.

## Introduction

Lung cancer is the most common cause of cancer-related deaths worldwide. The reduction of tobacco consumption is currently the major strategy to reduce lung cancer burden. However, identification of genes involved in causing the disease could contribute to discovering the underlying mechanisms and lead to a comprehensive prevention strategy as well as targeted therapy [1]. Some individuals are more susceptible to lung cancer than others and knowledge for the explanation for this inter-individual divergence in susceptibility is important for the purpose of developing methods that can predict the risk [2]. Although common genetic variants involved in lung cancer have been identified by genome-wide association studies, detailed information about the genetic factors remains to be elucidated. It seems that rarer genetic variants are likely to account for most of the individual susceptibility [1]. The candidate gene approach, which deals with previously identified genes that are thought to participate in the pathophysiology of the disease, is still employed as a useful tool.

In this study prostaglandin-endoperoxide synthase 2 (PTGS2) and cytochrome P450, family 2, subfamily E, polypeptide 1 (CYP2E1) were selected as candidate genes susceptible to lung cancer based on their critical involvement in the mechanism of lung carcinogenesis [3], [4]. *PTGS2* is a highly inducible gene, activated by cytokines, growth factors, oncogenes and chemical carcinogens [5]. Functional studies suggest that PTGS2 plays a role in carcinogenesis and is overexpressed in many human malignancies [6], [7], especially in lung adenocarcinoma and squamous cell carcinoma [8], [9]. CYP2E1 is an ethanol- and drug-metabolizing enzyme that can activate procarcinogens and hepatotoxicants, and generate reactive oxygen species [10], [11]. In addition, CYP2E1 can activate human carcinogens, including benzene and N-nitrosamines found in cigarette smoke [12], [13]. Due to these important functions, PTGS2 and CYP2E1 genes were investigated as logical candidates for lung cancer susceptibility.

Many studies have evaluated the association of polymorphisms in PTGS2 and CYP2E1 genes with lung cancer; however, the results have been inconsistent, and the biological effects based on statistical reasoning are still elusive. Given the importance of ethnic divergences in the genetic effects on complex diseases and the fact that genetic markers for proposed gene-disease associations vary in frequency across the populations, we evaluated five extensively studied polymorphisms in *PTGS2* (rs689466, rs5275, rs20417) and *CYP2E1* (rs2031920, rs6413432) genes in a large northeastern Chinese population to examine their associations, both individually and in combination, with lung cancer risk.

## Methods

### Study population

This was a hospital-based case-control study involving a total of 1286 subjects from the northeastern of China (Harbin city, Heilongjiang province). All subjects were local residents of Han descent. There were study population included 684 patients clinically diagnosed as lung cancer and 602 age-matched cancer-free controls. The study was approved by the Ethics Committee of Harbin Medical University, and conducted according to the Declaration of Helsinki Principles. All subjects signed the written informed consent.

### Diagnostic criteria and demographic characteristics

Lung cancer was diagnosed by chest radiograph and either high resolution computed tomography (CT) or enhanced CT or PET-CT scan and was pathologically confirmed by biopsy. Clinical subtypes of lung cancer consisted of squamous cell cancer, adenocarcinoma, small cell cancer and unspecified lung cancer. Age and gender were recorded when the subjects were recruited. Body weight in kilograms and height in meters were measured to calculate the body mass index (BMI, kg/m^2^). The status of cigarette smoking and alcohol drinking was defined at the time of the survey. Smoking was categorized as never, ever or current smoking (at least one cigarette per day). Drinking was categorized as never, ever or current drinking. Here, current drinking referred to consumption of at least one alcoholic drink during the past 30 days.

### Genotyping

Blood samples (2 mL) were collected and genomic DNA was extracted from white blood cells using the TIANamp Blood DNA Kit (Tiangen Biotect [Beijing] Co., LTD). Genotyping was conducted using the PCR-LDR (ligase detection reactions) method by ABI 9600 system (Applied Biosystems, USA) [14]. Cycling parameters were as the following: 94°C for 2 min; 35 cycles of 94°C for 15 s; 60°C for 15 s; 72°C for 30 s; and a final extension step at 72°C for 5 min. For each polymorphism, two specific probes to discriminate the specific bases and one common probe were synthesized. The common probe was labeled at the 3′ end with 6-carboxy-fluorescein and phosphorylated at the 5′ end. The reacting conditions of LDR were: 94°C for 2 min, 20 cycles of 94°C for 30 s and 60°C for 3 min. After reaction, 1 µL LDR reaction products were mixed with 1 µL ROX passive reference and 1 µL loading buffer, and then denatured at 95°C for 3 min, and chilled rapidly in ice water. The fluorescent products of LDR were differentiated using ABI sequencer 377 (Applied Biosystems, USA).

### Statistical analysis

Comparisons between lung cancer patients and controls were conducted by unpaired t-test for continuous variables and by χ^2^ test for categorical variables. To avoid gross genotyping error, all polymorphisms were checked for consistency with Hardy-Weinberg equilibrium on a contingency table of observed-versus-predicted genotype frequencies by using Pearson χ^2^ test or Fisher's exact test. Genotypes were compared by Logistic regression analysis under assumptions of additive, dominant and recessive models of inheritance, respectively. Statistical significance was defined as *P*<0.05.

Haplotype frequencies were estimated by using the haplo.em program, and odds ratio (ORs) and 95% confidence interval (CI) were estimated by haplo.cc and haplo.glm programs according to a generalized linear model [15]. Furthermore, the haplo.score was used to model an individual's phenotype as a function of each inferred haplotype, which was weighted by their estimated probability to account for haplotype ambiguity. The haplo.em, haplo.glm, and haplo.score were implemented using Haplo.stats software (version 1.4.0) developed by the R language (http://www.r-project.org/). Study power was calculated using PS (Power and Sample Size Calculations) software (version 3.0).

Interaction analysis was conducted in the open-source MDR software (version 2.0) (www.epistasis.org) [16], [17]. All possible combinations of one to four polymorphisms were constructed using MDR constructive induction. Then a Bayes classifier in the context of 10-fold cross-validation was employed to estimate the testing accuracy of each best model. A single best model had maximal testing accuracy and cross-validation consistency, which measures the number of times of 10 divisions of the data that the best model was found. Statistical significance was evaluated using a 1000-fold permutation test to compare observed testing accuracies with those expected under the null hypothesis of null association. Permutation testing corrects for multiple testing by repeating the entire analysis on 1000 datasets that are consistent with the null hypothesis.

## Results

### Baseline characteristics

The demographics and risk factors in the study population are summarized in Table 1. Cases and controls were well matched by age. Male gender, smoking, drinking and higher BMI level were associated with increased risk for lung cancer. Among all lung cancer patients, the subtype of adenocarcinoma, squamous cell cancer, small cell cancer, and unspecified cancer accounted for 37.54%, 32.26%, 20.83%, and 9.38%, respectively.

**Table 1:** The baseline characteristics of study population.

Characteristics	Patients (N = 684)	Controls (N = 602)	P*
Age (years), mean ± SD	57.24±9.84	56.80±9.95	0.776
Gender (male) (%)	72.78	66.49	0.013
BMI (kg/m^2^), mean ± SD	22.9±3.25	21.87±2.57	0.032
Smoking (%)			<0.005
Current	28.22	6.99	
Ever	8.04	0.54	
Never	63.74	92.47	
Drinking (%)			<0.005
Current	15.23	5.38	
Ever	1.61	2.69	
Never	83.16	91.94	
Lung cancer type (%)			
Squamous cell cancer	32.26	NA	
Adenocarcinoma	37.54	NA	
Small cell cancer	20.83	NA	
Unspecified	9.38	NA	

*Abbreviations:* BMI, body mass index; NA, not available.

* *P* values were calculated by using unpaired t-test for age and BMI, and by χ^2^ test for categorical variables.

### Single-locus analysis

With regard to rs20417, the frequency of mutated homozygote was zero in this study population, although its minor allele frequency was estimated at 7% in Beijing Han Chinese, and 41% in Nigerians (HapMap database). The genotype distributions of the other four polymorphisms followed Hardy-Weinberg equilibrium in control groups (*P*>0.05). As shown in Table 2, significant difference between lung cancer patients and controls was observed in allele and genotype distributions of rs5275 (P_allele_ = 0.002 and P_genotype_ = 0.003) and rs6413432 (P_allele_ = 0.037 and P_genotype_ = 0.044), and in genotype distributions of rs689466 (P = 0.02). Based on power calculation, the present study of 684 patients and 602 controls had 83.8% power to detect a significant association for rs5275.

**Table 2:** The genotype distributions and allele frequencies of the studied polymorphisms between patients and controls, and their risk predictions for lung cancer under three genetic models of inheritance.

Polymorphism	Patients (N = 684)	Controls (N = 602)	P_χ2_	Genetic models	OR; 95% CI; P
rs689466 AA	230	161		Additive	0.87; 0.74–1.01; 0.07
AG	318	320	0.02	Dominant	0.72; 0.57–0.92; 0.008
GG	136	121		Recessive	0.99; 0.75–1.3; 0.923
G (%)	43.13	46.68	0.071	Study power: 43.9%	
rs5275 TT	486	389		Additive	0.73; 0.6–0.9; 0.002
TC	185	181	0.003	Dominant	0.75; 0.59–0.95; 0.017
CC	15	32		Recessive	0.4; 0.21–0.74; 0.004
C (%)	15.72	20.35	0.002	Study power: 83.8%	
rs2031920 CC	403	361		Additive	1.02; 0.84–1.23; 0.855
CT	250	211	0.82	Dominant	1.04; 0.84–1.31; 0.702
TT	31	30		Recessive	0.91; 0.54–1.51; 0.704
T (%)	22.81	22.51	0.857	Study power: 5.4%	
rs6413432 TT	472	377		Additive	0.82; 0.68–0.99; 0.042
TA	182	198	0.044	Dominant	0.75; 0.6–0.95; 0.016
AA	30	27		Recessive	0.98; 0.57–1.66; 0.931
A (%)	17.69	20.93	0.037	Study power: 54.8%	

*Abbreviations:* OR, odds ratio; 95% CI, 95% confidence interval.

Notably, for rs5275, the risk associated with mutant allele or genotype was decreased by 27% (95% CI: 0.6–0.9; *P* = 0.002) under the additive model, by 25% (95% CI: 0.59–0.95; *P* = 0.017) under the dominant model, and by 60% (95% CI: 0.21–0.74; *P* = 0.004) under the recessive model. With regard to rs689466, carriers of the mutant G allele had a 28% reduced risk of developing lung cancer (95% CI: 0.57–0.92; *P* = 0.008) compared with the AA carriers (Table 2). A significant difference was identified for rs6413432 in association with lung cancer under the additive (OR = 0.82; 95% CI: 0.68–0.99; *P* = 0.042) and dominant (OR = 0.75; 95% CI: 0.6–0.95; *P* = 0.016) models. No significant association was detected for rs2031920 under the three genetic models.

### Genotype-phenotype analysis

In view of the differences in anthropometric characteristics between lung cancer patients and controls, it was of interest to investigate their correlation with the studied polymorphisms. We observed significant association between rs5275 and gender (χ^2^ = 6.07; *P* = 0.048), as well as a borderline association of rs5275 with smoking (χ^2^ = 5.16; *P* = 0.066). No significant association of other polymorphisms with anthropometric characteristics was observed (data not shown).

### Haplotype analysis

Haplotype frequencies of the four polymorphisms examined were estimated and compared between cases and controls (Table 3). The frequency of haplotype A-T-T-T (in order of rs689466, rs5275, rs2031920 and rs6413432) was significantly higher (*P* = 0.02) in patients than that in controls after statistical correction, whereas the frequency of haplotype G-C-C-T was significantly lower (*P* = 0.034) in patients. After assigning the commonest haplotype A-T-C-T as the reference, haplotype G-C-C-T decreased the odds of lung cancer by 28% (95% CI: 0.51–0.93; *P* = 0.019, study power = 97.7%) after adjusting for age, gender, smoking and drinking. In contrast, haplotype A-T-T-T had a 1.49-fold (95% CI: 1.21–1.79; *P* = 0.012, study power: 82.1%) increased risk.

**Table 3:** Haplotype frequencies of the studied polymorphisms between patients and control, and their risk prediction for lung cancer after adjusting for confounders.

Haplotype*	Patients (%)	Controls (%)	P_Sim_	OR; 95% CI; P†
A-T-C-T	37.47	36.61	0.206	Reference group
A-C-T-A	2.0	3.07	0.744	0.8; 0.41–1.56; 0.505
A-T-C-A	4.94	4.01	0.191	1.43; 0.82–2.49; 0.208
A-T-T-A	4.96	3.56	0.193	1.03; 0.71–1.51; 0.86
A-T-T-T	9.92	6.77	0.02	1.49; 1.21–1.79; 0.012
G-C-C-T	9.33	14.38	0.034	0.72; 0.51–0.93; 0.019
G-T-C-A	6.45	5.42	0.869	1.23; 0.71–2.14; 0.459
G-T-T-A	3.58	4.45	0.193	1.01; 0.62–1.66; 0.938
G-T-T-T	6.03	4.71	0.249	1.37; 0.6–3.12; 0.456

*Abbreviations:* OR, odds ratio; CI, confidence interval; P_Sim_, simulated P.

* Alleles in haplotype were presented in order of polymorphisms rs689466, rs5275, rs2031920 and rs6413432.

† *P* was calculated after adjusting for age, gender, smoking and drinking status.

### Interaction analysis

An exhaustive MDR analysis that evaluated all possible combinations of four studied polymorphisms is summarized in Table 4. Each best model was accompanied with its testing accuracy, cross-validation consistency and significant level as determined by permutation testing. The overall best MDR model included rs5275, rs689466, and rs6413432 polymorphisms. This model had a maximal testing accuracy of 66.1% and a maximal cross-validation consistency of 10 out of 10. This model was significant at the 0.003 level, indicating that a model this good or better was observed only by three times out of 1000 permutations and was thus unlikely under the null hypothesis of null association.

**Table 4:** MDR analysis summary.

Best combination of each model	Cross-validation consistency	Testing accuracy	*P*
rs5275	7	0.591	0.064
rs5275, rs689466	10	0.629	0.029
rs5275, rs689466, rs6413432	10	0.661	0.003*
rs5275, rs689466, rs6413432, rs2031920	10	0.646	0.017

* The overall best MDR model.

## Discussion

In this study we found significant associations of *PTGS2* and *CYP2E1* polymorphisms with the susceptibility to lung cancer in northeastern Chinese. Moreover, as implicated by haplotype and interaction analyses, we found potentially synergistic effect between these two genes, which reinforces single-locus results and contributes to strong susceptibility to lung cancer. To our knowledge, this is the first case-control study investigating the joint effect of *PTGS2* and *CYP2E1* genes on lung cancer.

Although the candidate gene approach cannot replace the genome-wide association study in unraveling the genetics of complex traits, it remains an important alternative strategy, particularly in the context of adequate sample sizes, ethnic homogeneous populations, and solid biological evidence of the genes concerned. To generate robust data, it has been proposed that a large sample size involving >1000 subjects in each group is required [18]. Despite that only 684 patients and 602 controls were enrolled in this study, in view of wide divergence in genetic distributions, a priori power calculation indicated that this study had >80% power to detect the loci of realistic effect size. Moreover, our study subjects were ethnically homogeneous and local residents of Harbin city, a site where the prevalence of lung cancer is relatively high likely because of the indoor air pollution from the unventilated coal-fueled stoves [19]. In addition, genotypes of studied polymorphisms were in Hardy-Weinberg equilibrium in controls, suggesting the results are unlikely to be biased by genotyping errors or population stratification. Furthermore, selection of genes and polymorphisms was based on strong biological, genetic and epidemiological indications [20]–[23]. However, considering the complexity of lung cancer, our preliminary results should be considered as hypothesis needing to be tested in larger well-designed studies.

Recently, several global meta-analyses have summarized the individual findings on *PTGS2* and *CYP2E1* genes and confirmed the significantly protective effect of rs5275, rs2031920 and rs6413432 mutant alleles on lung cancer [4], [24], [25]. In the current study, we identified that carriers of mutant alleles of rs689466, rs5275 and rs2031920 were at remarkably reduced risk for lung cancer. To expand the findings, we reported a potential synergism between *PTGS2* and *CYP2E1* genes on lung cancer risk, as reflected by haplotype and interaction analyses. Despite the marginal association of rs6413432 in single-locus analysis, compared with haplotype A-T-T-A (in order of rs689466, rs5275, rs2031920 and rs6413432), the risk magnitude associated with haplotype A-T-T-T was remarkably augmented and was independent of traditional risk factors, demonstrating a contribution of rs6413432-T allele to lung cancer susceptibility. However, in the presence of rs689466-G and rs5275-C alleles, the risk-conferring role of rs6413432-T allele was markedly attenuated showing a 28% reduced risk for haplotype G-C-C-T. Using the MDR method, which is nonparametric and genetically model-free in design [26], we constructed the overall best model encompassing polymorphisms rs5275, rs689466, and rs6413432 with strong synergistic effects. Since the pathophysiological mechanism underlying such interaction is as yet unknown, we speculate that *PTGS2* and *CYP2E1* genes might interact with each other to play a role in lung carcinogenesis. Furthermore, considering the divergent genetic profiles, such as the non-mutated rs20417 in our study population, it is necessary to establish an independent database of genetic markers for lung cancer in each ethnic group. Moreover, genotyping data from the *PTGS2* and *CYP2E1* genes, incorporating the haplotype and synergism analytical strategy would facilitate the identification of individuals at high risk of developing lung cancer in future clinical screening.

Some limitations should be considered when interpreting the results. First, the cross-sectional design of this study may preclude comments on causality, and a survival bias could not be excluded. Second, we only focused on five polymorphisms in *PTGS2* and *CYP2E1* genes and did not cover the whole genomic sequences of the genes, and thus we may under-evaluate the genetic effects of other genetic markers, Third, data on the circulating PTGS2 and CYP2E1 levels are unavailable, which makes us incapable of comparing their levels across genotypes. The immunocytochemical analysis of PTGS2 expression is ongoing to detect any genotype-phenotype association [27]. Fourth, the sample size of this study was relatively small such that our findings need to be validated in a larger population.

To sum up, our findings demonstrated a potentially synergistic effect between *PTGS2* and *CYP2E1* genes on the underlying cause of lung cancer in northeastern Chinese. Moreover, this study leaves open the question of divergent genetic profiles across different ethnic groups. This study provides supporting evidence for further investigation on pathophysiological mechanisms of *PTGS2* and *CYP2E1* genes in lung cancer.
